# Community collaboration to increase foreign-born women¿s participation in a cervical cancer screening program in Sweden: a quality improvement project

**DOI:** 10.1186/s12939-014-0062-x

**Published:** 2014-08-09

**Authors:** Erik Olsson, Malena Lau, Svante Lifvergren, Alexander Chakhunashvili

**Affiliations:** 1 Centre for Equity in Health/Kunskapscentrum för Jämlik Vård, Western Region of Sweden/Västra Götalandsregionen, Regionens Hus, Göteborg, SE-405 44, Sweden; 2 Centre for Healthcare Improvement and Division of Quality Sciences, Chalmers University of Technology, Göteborg, SE-412 96, Sweden; 3 Angered Local Hospital/Angereds Närsjukhus, Angered, Sweden; 4 Centre for Healthcare Improvement and Division of Quality Sciences, Chalmers University of Technology, Göteborg, SE-412 96, Sweden

**Keywords:** Cervical cancer screening, Community collaboration, Foreign-born, Native language, Doulas, Sweden

## Abstract

**Introduction:**

The prevailing inequities in healthcare have been well addressed in previous research, especially screening program participation, but less attention has been paid to how to overcome these inequities. This paper explores a key factor of a successful improvement project: collaboration with local *doulas* to raise cervical cancer screening participation by more than 40 percent in an area with a large number of foreign-born residents.

**Methods:**

Data was collected through two focus group discussions with the doulas in order to design interventions and debrief after interventions had been carried out in the community. Various tools were used to analyze the verbal data and monitor the progress of the project.

**Results:**

Three major themes emerged from the focus group discussions: barriers that prevent women from participating in the cervical cancer screening program, interventions to increase participation, and the role of the doulas in the interventions.

**Conclusions:**

This paper suggests that several barriers make participation in cervical cancer screening program more difficult for foreign-born women in Sweden. Specifically, these barriers include lack of knowledge concerning cancer and the importance of preventive healthcare services and practical obstacles such as unavailable child care and language skills. The overarching approach to surmount these barriers was to engage persons with a shared cultural background and mother tongue as the target audience to verbally communicate information. The doulas who helped to identify barriers and plan and execute interventions gained increased confidence and a sense of pride in assisting to bridge the gap between healthcare providers and users.

## Introduction

By the end of 2011, 15 percent of Sweden¿s population were born outside the nation¿s borders. In Gothenburg, the country¿s second largest city, the largest groups of foreign-born persons originated in Iran and Iraq [1]. The multicultural diversity is particularly evident in the northeast part of Gothenburg where almost 50 percent of the 100,000 residents are foreign-born. Over 40 languages are spoken in this part of the city, and besides Swedish, the most common languages are Arabic, Bosnian/Croatian/Serbian, Persian, Kurdish, Somali, and Finish. The poverty index and child poverty index are higher in northeastern Gothenburg than in the rest of the city, as are indicators of poor health such as physical inactivity, smoking, and obesity [2].

In Sweden, organized cervical cancer screening was implemented in the mid-1960s [3]. Since then, the screening programs have proved to significantly reduce the incidence of cervical cancer [4],[5]. Papanicolaou (Pap) smear testing offers early detection of precancerous cells and is included in Swedish screening programs. Following national recommendations [6], women in the Western Region of Sweden aged between 23 and 50 are invited to have Pap smear tests at an antenatal clinic every third year, compared to every fifth year for women aged between 50 and 60. Women may also choose to take the test at another location, such as their regular gynecological clinic. The question of whether HPV tests should replace Pap smears in the Swedish screening programs is studied [7]. Still, about 450 women are annually diagnosed with cervical cancer in Sweden, of which approximately 140 patients die [8]. In the Western Region of Sweden, over 80 percent of women between 23 and 60 participate in the screening program; however, in northeastern Gothenburg, the region¿s major city, only around 60 percent of the women participated in the screening program prior to this project [9],[10].

The Swedish Health and Medical Services Act [11] stipulates that the goal for Swedish healthcare is good health and care on equal terms for the entire population, including accessibility to services. Nevertheless, participation rates among foreign-born women are lower than among the Swedish-born in mammography screening [12] and cervical cancer screening [13]. International research indicates that fewer foreign-born women participate in screening programs for several reasons: unawareness of preventive healthcare services [14],[15]; difficulties comprehending the term *cancer* or fear of getting a cancer diagnosis [16],[17]; fatalistic attitudes or the belief that cancer has no cure [14],[17]; practical issues and administrative barriers [14],[16]-[19]; and language barriers [14],[18],[19].

Rather than written materials, oral dissemination of information could be important for mobilizing minorities to take Pap smear tests [14],[20]. In particular, engaging people from the same cultural background to inform a community has proved beneficial [17],[19],[21],[22]. In so doing, the perceptions and values of the community members are incorporated into the design of healthcare services, which in turn can better address community members¿ needs and increase the likelihood of successful health interventions [22],[23]. The common cultural background also creates credibility, visibility, and access to the population in need [24]. The benefits may not be only for the target audience, but also for the involved community messengers who experience a sense of self-efficacy as they make a difference in their community [25].

In this project, *doulas* were selected to represent the community. In northeastern Gothenburg, doulas support new parents during pregnancy and childbirth and have the same cultural background as those they support. Hence, their role is to interpret language as well as culture. In total, there are approximately 20 doulas in the area. Together they speak around 10 languages, the most common languages being Arabic, Somali, Persian, and Kurdish. The doulas were asked to participate in the project because they already had an established role in the community and previous experience working with healthcare providers. An evaluation of the doula project showed that the doulas¿ collaboration with healthcare staff could potentially create more equal distribution of healthcare [26].

The purpose of this paper is to explore how collaboration with community members in an area with a large number of foreign-born residents may contribute to increased participation in a screening program. The paper aims to elucidate barriers hindering women from participating in cervical cancer screenings and to identify interventions to overcome these barriers. Moreover, the paper also discusses the role of the doulas during the interventions.

## Methods

This project included collaboration with the doulas to address the problem of the low participation rate in the cervical cancer screening program and to identify and execute interventions to increase participation. Interventions were launched during one year and numerous meetings took place with various stakeholders. Central to this paper are two focus group discussions that were facilitated before and after the doulas executed service-improvement interventions in the community. An Ishikawa diagram was used to analyze the verbal data of the first focus group, and a control chart was used to monitor the number of Pap smear tests.

### Participants

To understand the needs and expectations of the women in the local context, two focus group discussions were conducted with the doulas. The first took place prior to their execution of interventions and focused mainly on barriers to participation and potentially successful ways to increase participation. The second was conducted in a more evaluative manner, focusing on the doulas¿ experiences in meeting women in squares and public places and in collaborating with healthcare personnel, mainly midwives. Four doulas participated in the first focus group discussion and nine in the second (Table 1), with three doulas participating in both. The four participants in the first group were selected because they were particularly active in the local area and were believed to have insight regarding barriers to local women taking the test. In the second focus group, all doulas were invited because the discussion centered on their experience in meeting with local women. Native language ability was an attribute of the doulas that was considered to be important to their outreach to the locals, especially since it was evident from the start that orally spread information in the locals¿ mother tongues would be a major activity to mobilize women to undergo Pap smear testing.

**Table 1:** The participants and native languages

**Language(s)**	**Number of participants**	
	**First focus group**	**Second focus group**
Arabic	3	2
Persian	1	1
Somali	0	4
Arabic and Persian	0	1
Arabic, Kurdish, and Turkish	0	1
**TOTAL**	**4**	**9**

### Data collection and analysis

Focus group discussion was chosen as a method because it allows a number of different voices to be collected simultaneously, but more important because group dynamics and relationships can be observed [27]. In this particular case, the method also was chosen because previous research suggests that community attitudes and patterns of behavior may be reproduced within focus groups and it is an appropriate method when participants come from cultures that draw on oral traditions, norms of helping, and existing social networks [28]. Moreover, the method is appropriate when developing culturally sensitive information [29].

EO and ML acted as facilitators in both focus group discussions that were conducted in Swedish. All of the doulas who participated in the discussions spoke Swedish; however, their fluency levels varied greatly, making it important to ensure that communication between doulas and the facilitators was understood by all parties. Both focus group discussions were held at the local hospital, a place the doulas new well and where they would be relaxed. All participants were informed about the purpose of the groups, that participation was voluntary, that the discussions would be tape-recorded, transcribed, and anonymized. Because the described project was a quality improvement initiative rather than a research project, no permission from the Ethics Committee was collected. The inquiry process was in line with the applicable principles as proposed by the Declaration of Helsinki [30]. The focus group discussions were analyzed using qualitative content analysis, based on the procedure explained by Graneheim and Lundman [31]. The transcriptions of the focus group discussions were read several times and coded. The various codes were compared based on differences and similarities and sorted into different categories. The categories were also compared and clustered under a number of emerging themes. In addition, an Ishikawa diagram [32] was used to analyze the verbal data of the first focus group and to identify root causes of the problem of low participation rates. The analysis helped to guide what interventions to prioritize and launch. Moreover, a control chart [32] was constructed to monitor the number of tests on a monthly basis.

## Results

In this section the results from the two focus group discussions are presented. Prior to the first focus group, the doulas were given introductory training from a midwife to prepare them to meet the public and to answer questions about Pap smear tests and the screening program.

### Identifying barriers and designing interventions

In the spring of 2011, the doulas were invited to a focus group discussion that concerned barriers hindering local women from taking the Pap smear test. During the discussions, interventions to increase participation in the cervical cancer screening program also were considered. The group discussed the doulas¿ roles in communicating with women in the community pertaining to cervical cancer prevention.

The doulas agreed that the main reason women in the local area did not participate in the cervical cancer screening program was simply because they did not know it existed or did not understand the purpose. Most women had not even heard about Pap smear tests, one doula said. The doulas said that some women had even taken the test but still did not understand its purpose because no interpreter had been present to explain. Written information was insufficient, and if translated, was still hard to understand. Often the women¿s children acted as translators, although the doulas believed that this kind of information was too hard for children to translate. Some women knew about the screening program and the test, but simply ignored the screening invitations because they thought they could not spare the time away from their children. Another reason to avoid the test was fear, both of the awkward test situation and also of cancer. The doulas believed that many women with the same cultural backgrounds as themselves thought cancer could be neither prevented nor cured. From her experience, one doula understood the reason why some women decided not to take the test:

We have family history of cancer. I was frightened of mammography and said I did not want to know whether I was sick or not ¿ By information I have received I feel ¿ but I have not yet done it ¿ that I would like to undergo mammography as well as taking a Pap smear test. I want to know if I have it or not.

Because many local women were unfamiliar with preventive healthcare services, the doulas believed it was important to explain Pap smear testing carefully. One doula thought that newly immigrated women must ¿get into the system of prevention¿ quickly or else they would ignore it and risk seeking care too late.

They think it is the same thing as with the dentist: ¿I go there and open my mouth and then I pay for nothing ¿ if I lose a tooth or have a hole in a tooth and I can¿t eat, then I would go to the dentist¿.

The doulas believed the patients¿ fee (approximately 10 euros) was too low to pose a barrier. One doula believed that Pap smear tests could be taboo to discuss because the test has to do with women¿s private parts, which women did not talk about even with people they knew. However, most of the doulas thought they could talk about the Pap smear test to most local women in their mother tongues because the doulas together spoke many languages. They also believed that they could help women to understand the screening invitations and other written information from healthcare providers. If child care were unavailable, one doula thought that they could help look after the children when the women took the test. Because of the doulas¿ already established role in the community regarding pregnancy and childbirth issues, the local women would trust them to look after their children.

In their role as doulas, they believed that they had good access to various area associations, which could serve as platforms for informing women because these groups already gathered people to talk and discuss things. Of course, this kind of information also could be given in churches, adult education, or Swedish classes, the doulas said. One doula had suggested to a Swedish language teacher that the students practice their language skills by talking about Pap smear tests, and the teacher¿s response had been positive. Another doula believed that information should be given in high schools. Even though female students were too young to participate in the screening program the information would prepare them when their screening invitations arrived a few years later. They also believed that they should take advantage of local events and seize the opportunity when a large number of the community members were gathered. Similarly, the doulas could provide information at the clinic where the women took the test to make sure that at least those who *were* tested understood the reason. The doulas said they would prefer to work in pairs with mixed language skills to make sure they would get the message across to as many women as possible. The doulas believed that once they got started, the word would spread from mouth to mouth.

This woman, I am sure, will spread the word to other women, if she knows what it is, and the other woman will talk about it here and there. If only one person understands, everybody will know.

The doulas held different opinions about whether information should be given in groups with both men and women present. Some doulas thought that it would be easier to ask questions in groups of women only. Another agreed, but thought that there could be certain things women and men could be informed about together and other things that were too sensitive for a mixed group. One doula said that men sometimes were the ones who prevented their wives from taking the test. Maybe if men got information too, they would talk about it with other men and it would ¿become something normal, nothing strange¿. One doula was positive about inviting men to discussions about what at first glance seemed to be a women¿s issue:

Regarding men and women, we have experience about it because we are the first that had men in our group for moms and we talked a lot about different things and the men were very positive, very active. They would like to learn and they did know a lot ¿ They want to support their wives in good and bad.

**Figure 1:**
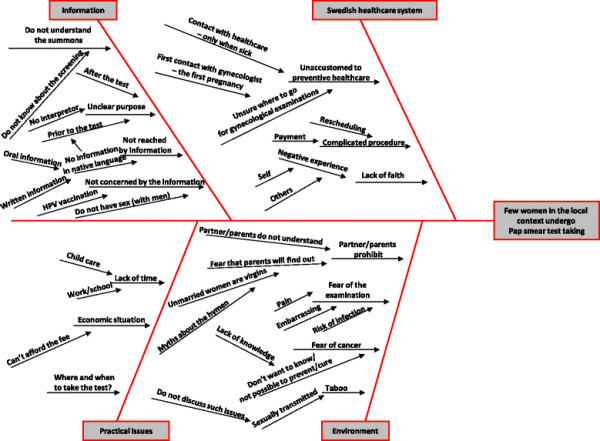
Ishikawa diagram.

Based on the first focus group discussion, an Ishikawa diagram [32] was constructed to analyze the barriers to taking the Pap smear test in the local context (Figure 1). As the project proceeded, barriers in the diagram were rejected or confirmed, depending on the doulas¿ and midwives¿ stories as they met the local women. As shown in Figure 1, four main dimensions were identified as hindering local women from taking the Pap smear tests: *information*, such as materials not being understood or available; *the Swedish healthcare system*, such as being unfamiliar with provided services; *practical issues,* such as lack of time or not knowing where to take the test; and *environmental* explanations, such as fear of the examination or taboos.

Other than the different views on whether information about Pap smears should be given to men, the doulas seemed to agree about the discussed barriers and interventions in the focus group. The identified barriers were a major resource that informed the interventions that were launched in the subsequent year.

In spring 2011 the doulas began to make presentations at local events and association meetings. As they encountered questions they could not answer, they received follow-up training from a midwife. The doulas worked together with midwives when presenting information through associations and outreach activities, such as the use of a mobile unit for Pap smear testing. After one year, the project was officially complete and the number of Pap smear tests in the area had increased by 42 percent compared with the previous year. According to the Swedish Personal Data Act [33], registering data that reveals race or ethnicity is prohibited. Hence, the distribution of the increase across different population groups based on the above characteristics could not be tracked.

### Debriefing after the interventions

After the interventions, a second focus group discussion was conducted with the doulas. This discussion took an evaluative approach, focusing on how the interventions had worked in the field, if the barriers had been accurately identified, and on the doulas¿ experiences.

The doulas believed they had largely succeeded in their primary mission: creating knowledge about Pap smear tests in the local community. The written information was obviously insufficient because word-by-word translations did not get the message across. The doulas were confident that the target audience understood the message better through discussion than reading printed information. They said that effective messengers had to know where to communicate the message and how to speak the women¿s mother tongues. Moreover, when women were fearful of the test, the doulas were careful to stress the importance of early detection to prevent cancer. They also told the women that they cared about their well-being. In sum, the doulas believed that because they were the ones sharing the information, women decided to take the test.

When they see us, and recognize us, they feel safe to ask about it.

The doulas also said they had a feeling of satisfaction when they could convince women the importance of being tested. Once they had communicated the message clearly, they believed the women would probably take the test regularly. The doulas also reported that they were able to reach women who had never considered visiting the clinic.

We reached women that had lived in Sweden for more than 15 years and never had the test taken ¿ These women were very happy and grateful afterwards since they did not dare before.

By participating in local events and through associations, the word spread and the doulas received an increasing number of questions from community members. Most doulas lived in the area and could answer questions around the clock. They knew the people well and engaged business owners as ¿partners¿, for example, local fruit stores, computer shops, kebab shops, and hair salons. Store owners ¿ men as well as women ¿ allowed the doulas to post stickers with information and sometimes to inform their customers about the importance of the Pap smear test. Two doulas even looked after the shop while the owner went to take the test. Talking about a hair salon, one doula said:

¿ She [the hair dresser] really has a great impact. People hang around there even if they don¿t cut their hair. They go there to drink coffee and to talk. She informed everybody there.

The intervention that created the most attention was the mobile unit, a bus with facilities set up to offer the Pap smear test in local squares and public places. The doulas believed the mobile unit was a positive intervention, although some things could have been done differently. They said the bus could have been parked more discretely to avoid the most crowded places. The doulas were able to look after the women¿s children during the testing at the mobile unit, but suggested having toys available for the children. The doulas reported that the mobile unit offered the test at no charge, which attracted women for whom the fee had been a barrier. The bus created a lot of attention and women, men, and children were curious and asked the doulas a lot of questions. Some women did not take the test right away, but returned with a friend a few days later. A few of the doulas reported that some men had approached them and made offensive remarks; for instance, one accused the doula of spreading disease. However, another doula had a positive experience in informing men about the Pap smear test through the mobile unit:

It was interesting that men approached me and asked about the test. After a while they came back with their wives.

In addition to talking about Pap smear tests and cervical cancer, the doulas also discovered other topics that people had questions about, such as mammography, the human papillomavirus (HPV) vaccination, contraception, and prostate cancer. The doulas believed that similar interventions should be carried out to raise awareness about such topics. They also reported that foreign-born women were not the only ones who had not had a Pap smear test; many Swedish-born women also had never had the test. Thus, they identified a need to focus on all women in the future.

The doulas said their collaboration with healthcare staff, mainly midwives, had been positive and they had no problem calling the midwives for information if questions arose that they could not answer. The doulas appreciated a midwife¿s training sessions because the doulas wanted to understand the information thoroughly before informing community members. Some doulas had not known much about the Pap smear test prior to the project. One doula said that she had taken the test ¿just in case¿, but she had not understood the test¿s purpose until she took the midwife¿s training. Not only did the doulas believe they had learned a lot, they also felt that they had done something important. The doulas¿ experiences in the interventions were mostly positive, and they enjoyed trying new ways of working. The doulas said they had become more confident in talking to people and were more sure of what to say.

In the beginning it was hard to approach people and talk to them. Some people were very open, but others very closed and said ¿no thank you.¿ Eventually it was great!

**Figure 2:**
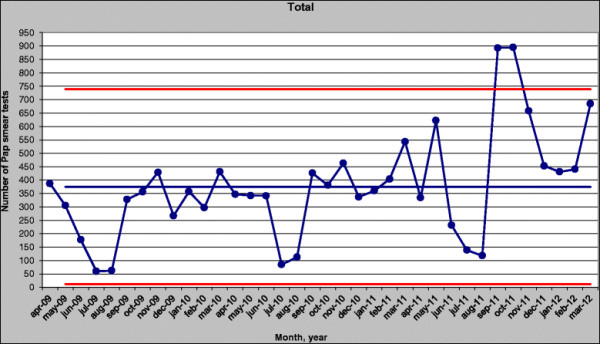
Control chart.

The doulas informed the community through events, associations, and outreach activities during one year. The number of Pap smear tests done per month were monitored on a control chart [32] to track the effect of the interventions (Figure 2). The chart included data from April 2009 through March 2012, the last month of the interventions. As shown in Figure 2, the number of tests increased by an average of approximately 200 per month during the intervention period (April 2011 to March 2012) compared with the period before the intervention (April 2009 to March 2011). This number reached its peak in September and October 2011, when the tests nearly tripled compared with the period before the intervention. The numbers for these two months fall beyond the upper control limit, computed as the center line (mean of the process), plus three times the process standard deviation. Therefore, they can indicate an assignable cause of variation in the desired direction, which confirms the positive effect of the intervention during this period ¿ primarily the mobile unit and the doulas¿ intensified activities.

## Discussion

The purpose of this paper is to explore how community participants in an area with a large number of foreign-born residents may improve use of preventive healthcare services. The paper presents not only barriers to women¿s participation in a cervical cancer screening program and interventions to overcome these barriers, but also the role of the community participants themselves during the interventions.

Discussions of *foreign-born* women can be complicated because these community members constitute a heterogeneous group from different continents and with different languages. Their reasons for migrating to Sweden, their age at migration, and their duration in Sweden vary, giving them different experiences. However, they also have similarities. Because these women were *not* born in Sweden and have mother tongues other than Swedish, they may find the Swedish healthcare system difficult to understand. Previous research suggests that women who attend cervical cancer screening programs are socialized into accepting these services to a greater extent than nonattendees [34]. The longer the duration in the new country, the greater chance of attending screening programs [35], suggesting that many foreign-born women may be socialized into accepting screening programs.

### Barriers and interventions

Many of the identified barriers in the first focus group discussion were confirmed by the doulas in the second focus group held after interventions took place. These findings align with previous research. Ignorance about preventive healthcare services proved to be a major barrier [14],[15]. Some women had difficulties comprehending the term *cancer* or were fearful of getting a cancer diagnosis [16],[17]. Also practical issues, administrative barriers [14],[16]-[19], and language [14],[18],[19] hindered women in northeastern Gothenburg from taking the test. Unlike previous research, fatalistic attitudes [14],[17], religious beliefs [18], or female genital mutilation [14] were not mentioned in the focus group discussions. The absence of the latter barrier may be due to the fact that no doula from a country in which female genital mutilation occurs was present in in the first focus group.

As with previous research, oral dissemination of information [14],[20] and communication by key actors who shared the audience¿s cultural background [17],[19],[21],[22] seemed to have been important in this project. A positive effect of the doulas¿ participation was that the community got involved. The doulas included their existing networks as they enlisted shop owners and associations as partners. Although the primary target population was women ages 23 to 60, the curiosity of other local residents should not be underestimated. During the project ¿ and particularly with outreach activities such as the mobile unit ¿ men and children also approached the doulas to get information about the test. Similar to previous research [36], male community members should also be included in educational efforts regarding cervical cancer prevention. Oral communication among people in this area of the city possibly had a major impact on the positive results. This finding aligns with previous research in which community representation created a greater diffusion of health knowledge in the community [37].

### The role of the participants

The doulas¿ common cultural backgrounds with the community created credibility [24] that not only facilitated their communication with the women they met, but also allowed them to receive information they could report back to healthcare providers. In this way, the doulas were able to illuminate the need for information about mammography, prostate cancer, and other medical issues, and to discover that Swedish-born women also needed information about Pap smear testing. Naturally, other factors besides place of birth ¿ such as age, educational level, socioeconomic status, and so on ¿ may effect participation in screening programs. However, these aspects were not the focus of this project.

Screening programs may be perceived as impersonal and anonymous [38]; therefore, the doulas¿ visibility [24] also may have had a positive effect on the foreign-born population¿s participation in the healthcare system. However, giving the doulas such visible role was not without risk. Though infrequently reported and as in previous research [24], some community members disliked the work of the doulas. But for the most part, the community positively embraced the doulas, who took a lot of pride in the project¿s positive results. They reported that the experience had made them more confident and that they had learned a lot themselves. As reported in previous research, the doulas clearly felt they had made a contribution and had played an important role in the healthcare system [25].

Based on the focus group discussions, the doulas functioned as one group regardless of language or origin. The mix of languages was seen as an advantage when working together. In the focus groups, the doulas gave each other praise and support for things like being calm when encountering rudeness and showing patience when women did not understand the message. Since most of the doulas lived in the community, they worked as informants even beyond their paid hours [24], and they did not report this extra effort as something negative. Although it is impossible to guarantee that communication involving community members will be completely accurate and value-free [24], the doulas were given training before and during the interventions and the opportunity to ask a midwife when they faced questions they could not answer. Overall, the collaboration between doulas and midwives proved successful and complemented each other¿s competence. The midwives brought their healthcare-related competence to the project and the doulas offered their cultural specific competence and local knowledge. The fact that the doulas already had an established role in the society proved to be a key factor for successful dissemination of information.

The doulas¿ representation of the local community may be questioned; after all, only five of the more than 40 languages spoken in the area [2] were represented in the focus group discussions. Some languages were not represented within the existing organization of doulas. Unfortunately, the only Bosnian/Croatian/Serbian-speaking doula did not participate in any of the focus group discussions, omitting one of the most commonly spoken languages in the area. The representativeness of the doulas also may be problematic because all were rather well integrated into the Swedish society and spoke Swedish. The doulas¿ experiences may have been too distant from those believed to be the hardest to reach, women who do not speak Swedish and have no or little knowledge of the Swedish healthcare system. Prior to the interventions the doulas did not believe that the patients¿ fee was a barrier, but they learned just the opposite from their experiences in the community ¿ the fee was a barrier for some women. Perhaps this disparity stemmed from the doulas¿ having a better financial status through working than some other women in the area. Despite these risks, the doulas¿ established role was more an advantage than a disadvantage. The doulas were believed to possess unique local and culturally specific knowledge and skills about where and how to inform the local women. However, their role did not stop with providing information; they also were able to receive information about community needs and expectations.

The sustainability of the project is continuously being evaluated. Two years after the project had ended, participation rates remained at the same high level as they had been during the project year. The experiences from the project have been transferred to an annual weekly campaign in which midwives and doulas continue to collaborate around information about cervical cancer prevention. This weekly campaign has also spread to other parts of Sweden.

## Conclusion

This project is a consequence of horizontal inequity, in which people with equivalent needs do not have access to the same resources [39],[40]. In this particular case, the need was defined as the knowledge to make an active choice for one¿s own health, that is, understanding the reason to undergo Pap smear testing. However, accessibility to resources or preventive healthcare services is not the same for all groups. Indeed, services and information about them are delivered in such a way that not all members in society can make an active choice for their own health. Often, an ethical dimension of justness and fairness is included in the concept of inequity [41]-[43]. Applied to this case, equal distribution of information to all subgroups in society cannot be considered fair or just because they face different barriers and respond to different forms of information. This project highlighted the importance of adapting solutions to the needs and expectations of a particular subgroup in order to increase equity.

Collaborating with community participants to identify barriers to healthcare services is necessary in order to design culturally specific interventions that are more likely to meet the various needs of the local population. But collaboration should not stop there ¿ executing the interventions also should involve the participants. Doing so, the information may be better suited to meet cultural expectations, and the information flow also can be reciprocal as healthcare providers receive requests from locals for other information. The community members involved may benefit from such participation themselves, such as increased confidence and a sense of pride and a smaller gap between healthcare providers and users.

The findings in this paper suggest that more research is needed about community participants¿ involvement in (re)designing outreach programs and how their role may be affected by such participation.

## Competing interests

The authors declare that they have no competing interests.

## Authors¿ contributions

EO and ML were managing the project, carried out data collection and analysis and revised the manuscript. EO drafted the manuscript. SL proposed methods for the project and helped with revisions of the manuscript. AC constructed the control chart and wrote the section about it. All authors read and approved the final manuscript.
